# Quantification of Peripheral Blur in Ultra-Widefield Fundus Imaging Following Diffractive Multifocal Intraocular Lens Implantation

**DOI:** 10.3390/jcm15156044

**Published:** 2026-08-03

**Authors:** Seong Min Kim, Seung Pil Bang, Seung-Bo Lee

**Affiliations:** 1 Department of Medical Informatics, Keimyung University School of Medicine, Daegu 42601, Republic of Korea; 2 Department of Ophthalmology, Keimyung University School of Medicine, Daegu 42601, Republic of Korea

**Keywords:** ultra-widefield fundus imaging, multifocal intraocular lens, image sharpness, peripheral blur, chromatic aberration, cataract surgery

## Abstract

**Background/Objectives:** Diffractive multifocal intraocular lenses (IOLs) are widely used following cataract surgery, but their impact on peripheral retinal image quality in ultra-widefield (UWF) fundus imaging has not been objectively characterized. This study aimed to quantify peripheral image sharpness in UWF fundus photographs and assess wavelength-specific differences between diffractive multifocal and enhanced monofocal IOLs. **Methods:** In this retrospective cohort study, 65 eyes from 51 patients implanted with enhanced monofocal (Tecnis Eyhance; *n* = 32) or diffractive multifocal IOLs (Tecnis Synergy, *n* = 19; Clareon PanOptix, *n* = 14) were analyzed. Non-mydriatic UWF fundus photographs (Optos; ~200°) were acquired within 1 month postoperatively. Red (R), green (G), and composite channel images were analyzed using three sharpness metrics—Laplacian variance, Brenner gradient, and Tenengrad gradient—within a predefined peripheral annular region (1.5–3.0 times the fovea–optic disc distance). Generalized estimating equations (GEE) with age as a covariate were applied as the primary analysis to account for inter-eye correlation and age differences between groups. **Results:** Brenner and Tenengrad gradient scores were significantly lower in the multifocal group in the R channel (*p* = 0.037 and *p* = 0.041, respectively), but not in the G or composite channels. The Laplacian metric revealed no significant intergroup differences in any channel. Subgroup analysis revealed no significant difference between hybrid multifocal and quadrifocal subtypes (*p* > 0.05). Group-level mean heatmaps confirmed a spatially distributed reduction in gradient magnitude across the annular zone in multifocal eyes. **Conclusions:** Diffractive multifocal IOL implantation was associated with a localized reduction in peripheral R channel sharpness, hypothesized to reflect transverse chromatic aberration. Composite channel quality remained comparable between groups, suggesting limited impact on routine clinical imaging. These findings may inform IOL selection and interpretation of peripheral retinal findings in multifocal IOL patients.

## 1. Introduction

Cataract surgery is one of the most frequently performed surgical procedures worldwide and has evolved from simply restoring distance vision to optimizing visual performance through advances in intraocular lens (IOL) technology [1]. Diffractive multifocal IOLs have been widely adopted to enhance functional vision across multiple focal distances while reducing spectacle dependence, with numerous studies reporting excellent uncorrected visual acuity and high patient satisfaction [2,3].

Despite these advantages, concerns remain regarding image quality under conditions requiring high optical precision [4]. Retinal specialists have been relatively cautious in adopting diffractive multifocal IOLs, as diffractive optics can induce glare, halos, reduced contrast sensitivity, and peripheral image blurring that may interfere with precise visualization of the peripheral retina [5,6]. Such blurring is clinically relevant during retinal examination and vitreoretinal procedures, where accurate identification of retinal tears, ischemic changes, or subtle vascular abnormalities is essential [6].

Ultra-widefield (UWF) fundus imaging has become an indispensable modality in routine ophthalmic practice, allowing visualization of up to approximately 200 degrees of the retina and improving detection of peripheral retinal pathology compared with conventional fundus photography [7,8]. If image degradation associated with diffractive multifocal IOLs extends to non-mydriatic UWF fundus photographs, subtle but clinically significant peripheral findings may be obscured, potentially influencing diagnostic accuracy, treatment decisions, and longitudinal monitoring.

Although previous investigations of multifocal IOLs have primarily focused on central visual performance and subjective visual outcomes [2,3,5], objective quantitative assessment of peripheral image quality using wide-angle retinal imaging remains limited. It is unclear whether peripheral image sharpness differs between monofocal and diffractive multifocal IOLs under real-world UWF imaging conditions. Furthermore, because UWF imaging systems rely on wavelength-dependent scanning modalities [9], potential differences across imaging wavelengths (red, green, and composite) may have distinct clinical implications. However, such channel-specific effects have not been objectively quantified in eyes implanted with diffractive multifocal IOLs.

Therefore, the purpose of this study was to objectively evaluate peripheral image sharpness in UWF fundus photographs of eyes implanted with monofocal and diffractive multifocal IOLs using established quantitative sharpness metrics. By providing channel-specific and design-specific analyses, this study aimed to determine whether diffractive multifocal IOLs meaningfully affect peripheral retinal image quality. The findings may offer clinically relevant evidence to inform IOL selection and interpretation of UWF retinal imaging in daily practice.

## 2. Methods

### 2.1. Participants

The study adhered to the tenets of the Declaration of Helsinki and was approved by the Institutional Review Board of Keimyung University Dongsan Hospital (IRB No. 2026-03-066, date of approval: 29 March 2026), which waived the requirement for written informed consent because of the retrospective design.

A total of 81 eyes from 63 patients who underwent phacoemulsification cataract surgery at Keimyung University Dongsan Hospital between May 2024 and May 2025 were initially reviewed. Images were excluded if eyelid or finger artifacts occupied more than 10% of the peripheral analysis zone, as defined in the Comparison of Image Sharpness section, or if image quality was otherwise ungradable due to prominent acquisition artifacts (e.g., horizontal banding). Eyelash artifacts could not be reliably excluded, as their appearance on UWF imaging is visually similar to retinal structures and could not be robustly distinguished even by manual review. After applying these criteria, 16 eyes were excluded—15 due to eyelid or finger artifact coverage exceeding 10% of the peripheral analysis zone (monofocal: 8 eyes, multifocal: 7 eyes), and 1 due to ungradable image quality from a prominent acquisition artifact in the multifocal group. The final cohort comprised 65 eyes from 51 patients.

Eligible participants were adult patients aged 24 years or older who had undergone uncomplicated cataract surgery with implantation of enhanced monofocal or diffractive multifocal IOLs and had ultra-widefield (UWF) fundus photographs acquired within 1 month postoperatively. The source cohort consisted of eyes without coexisting retinal or optic nerve disease, prior intraocular surgery, or media opacity. Demographic and clinical information, including age, sex, and laterality, was obtained from electronic medical records.

In cases of bilateral cataract, both eyes were eligible and were analyzed as separate units when both met the inclusion criteria. Patients were classified into a monofocal IOL or multifocal IOL group according to the implanted lens type. In this study, enhanced monofocal IOLs were classified as monofocal IOLs for group comparison. Accordingly, the monofocal group included 32 eyes from 26 patients exclusively implanted with a Tecnis Eyhance IOL (Johnson & Johnson Vision, Santa Ana, CA, USA), whereas the multifocal group included 33 eyes from 25 patients implanted with presbyopia-correcting IOLs, including Tecnis Synergy (Johnson & Johnson Vision, Santa Ana, California, USA) and Clareon PanOptix (Alcon Laboratories, Fort Worth, TX, USA). For subgroup analysis, the multifocal IOL group was further categorized into two predefined subgroups based on optical design: hybrid multifocal IOLs (Tecnis Synergy) and quadrifocal IOLs (Clareon PanOptix) [10].

### 2.2. Image Acquisition and Preprocessing

UWF fundus photographs were acquired using an Optos fundus camera (Optos PLC, Dunfermline, UK) under standard clinical imaging protocols. All images had a uniform size of 3900 × 3072 pixels and provided an ultra-widefield view of approximately 200 degrees. Images were acquired under standardized non-mydriatic conditions, with patients seated in a dark room and instructed to maintain central fixation. For each eye, all available images corresponding to the red (R), green (G), and red-green composite channel images were included. The R and G channel images were initially grayscale and required no further conversion. The composite channel images were originally in color and were converted to grayscale prior to analysis to ensure consistency across channels. All images were exported in lossless format and processed using Python 3.9 with the OpenCV (v4.11.0) and NumPy (v2.0.2) libraries. No additional image preprocessing (e.g., resizing, intensity normalization, or noise filtering) was applied.

### 2.3. Image Sharpness Metrics

To evaluate peripheral image quality of ultra-widefield fundus images, three established sharpness metrics were used: Laplacian variance, Brenner gradient, and Tenengrad gradient. These metrics capture complementary aspects of image sharpness and provide an objective assessment of this property.

The Laplacian variance was used to capture second-order spatial intensity variations and was defined as the variance of the Laplacian-filtered image,

$$L\left(I\right)=Var(\nabla^{2}I\left(x,y\right))$$

where I(x,y) denotes the grayscale image intensity. Higher values indicate stronger high-frequency components and greater image sharpness [11].

The Brenner gradient measured first-order intensity changes and was calculated as the mean squared difference between horizontally shifted pixel pairs,

$$B\left(I\right)=\frac{1}{N}\sum_{x}\sum_{y}{\left[I\left(x+d,y\right)-I(x,y)\right]}^{2}$$

where d = 2 pixels and N is the number of valid pixels within the masked region [12].

The Tenengrad metric quantified edge strength using Sobel operators and was defined as the mean squared gradient magnitude,

$$T\left(I\right)=\frac{1}{N}\sum_{x}\sum_{y}[G_{x}^{2}\left(x,y\right)+G_{y}^{2}(x,y)]$$

where $G_{x}$ and $G_{y}$ denote the horizontal and vertical Sobel gradients, respectively [13].

### 2.4. Comparison of Image Sharpness

Image sharpness was compared between the monofocal and multifocal IOL groups within the predefined peripheral annular region. For region-based analysis, the fovea was used as the center, and the distance between the fovea and optic disc served as an internal scaling reference. Based on this distance, two concentric circular masks with radii of 1.5 and 3.0 times the fovea–disc distance were generated, defining a central region (≤1.5) and a mid-peripheral annular region (1.5–3.0) [14,15]. Throughout this study, we use the term ‘peripheral’ to refer specifically to the mid-peripheral/near-equatorial annular zone spanning 1.5–3.0 times the fovea–optic disc distance, rather than the far peripheral retina captured at the extreme edge of the ultra-widefield field. This region was selected because it is well outside the posterior pole yet is consistently captured with minimal eyelid artifact or peripheral distortion across all images, unlike the outermost field, which is more variably visualized.

All sharpness metrics were computed within the peripheral annular region. For each metric, peripheral sharpness scores were compared between eyes implanted with monofocal and multifocal IOLs separately for the R, G, and composite channels.

### 2.5. Statistical Analysis

Descriptive statistics, including medians and interquartile ranges (IQRs), were computed for peripheral image sharpness scores. Normality of the scores was assessed using the Shapiro–Wilk test. To account for inter-eye correlation and to adjust for the significant age difference between groups, generalized estimating equations (GEEs) with an exchangeable correlation structure were applied as the primary analysis, using patient ID as the clustering variable and age as a covariate. Analyses were performed across three predefined sharpness metrics and three channels, as specified a priori; unadjusted GEE-derived *p*-values are reported. For subgroup analysis, peripheral sharpness scores were compared between hybrid multifocal and quadrifocal IOL subtypes using the Mann–Whitney U test. Two-sided *p* values < 0.05 were considered statistically significant. Boxplots were generated to visualize the distribution of sharpness scores across lens types and channels. All statistical analyses were performed using Python 3.9 with the Scipy (v1.18.0) and Statsmodels (v0.14.6) libraries.

## 3. Results

### 3.1. Participant Demographics and IOL Distribution

Sixty-five eyes from 51 patients were analyzed (Table 1). Eyes were classified into monofocal and multifocal IOL groups. The multifocal IOL group was significantly younger than the monofocal IOL group (*p* < 0.01). No significant differences were found in sex (*p* = 0.62) or laterality (*p* = 0.62) between the groups. The monofocal group included 32 eyes, and the multifocal group consisted of Tecnis Synergy (*n* = 19) and Clareon PanOptix (*n* = 14).

### 3.2. Quantitative Comparison of Image Sharpness Metrics

**Figure 1:**
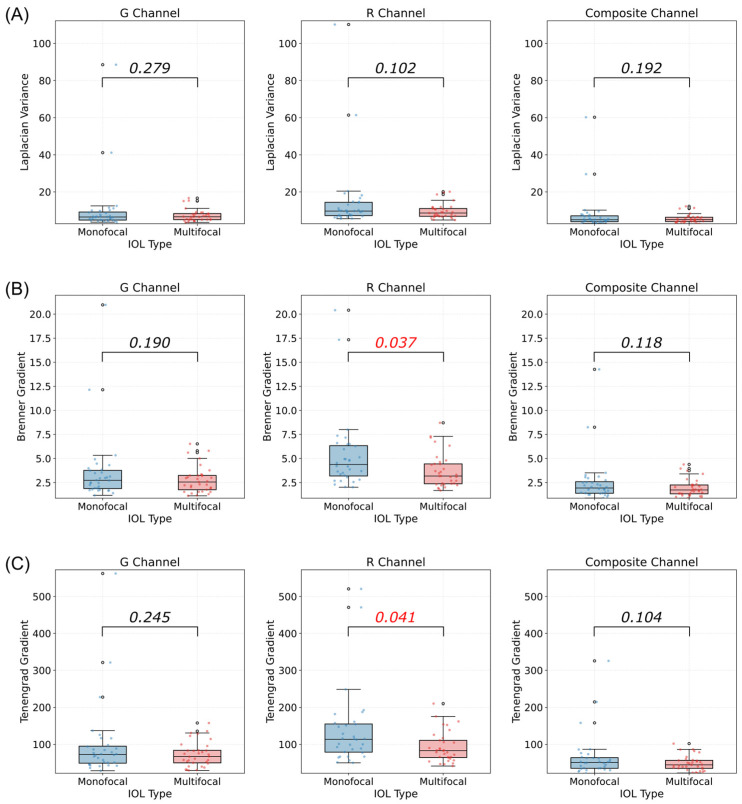
Comparison of peripheral image sharpness between monofocal and multifocal IOL groups. Boxplots illustrate peripheral sharpness distributions for the green (G), red (R), and composite channels for each metric: (**A**) Laplacian variance, (**B**) Brenner gradient, and (**C**) Tenengrad gradient magnitude. *p* values were derived from GEE analysis accounting for inter-eye correlation, with age as a covariate. Statistically significant differences are indicated in red.

Peripheral image sharpness was compared between monofocal and multifocal IOL groups using three established sharpness metrics across R, G, and composite channels (Figure 1). For the Laplacian metric, no significant differences were detected across any color channel, with nearly identical median values and overlapping IQRs between monofocal and multifocal groups (Figure 1A).

In contrast, first-derivative–based sharpness metrics demonstrated channel-specific group differences. For the Brenner metric, the multifocal group showed significantly reduced sharpness in the R channel compared with the monofocal group (*p* = 0.037), whereas no significant differences were detected in the G and composite channels (all *p* > 0.05) (Figure 1B). Tenengrad scores exhibited a similar pattern, with significantly lower R channel sharpness in multifocal eyes than in monofocal eyes (*p* = 0.041), and no significant differences in the remaining channels (all *p* > 0.05) (Figure 1C).

Overall, both Brenner and Tenengrad metrics consistently indicated a selective reduction in R channel peripheral image sharpness scores in multifocal eyes, a channel-dependent effect that was not captured by the Laplacian metric. Across all metrics, eyes in the monofocal IOL group tended to show higher upper-end outliers, although the frequency of outliers varied by metric and channel.

### 3.3. Spatial Distribution of Peripheral Blur

**Figure 2:**
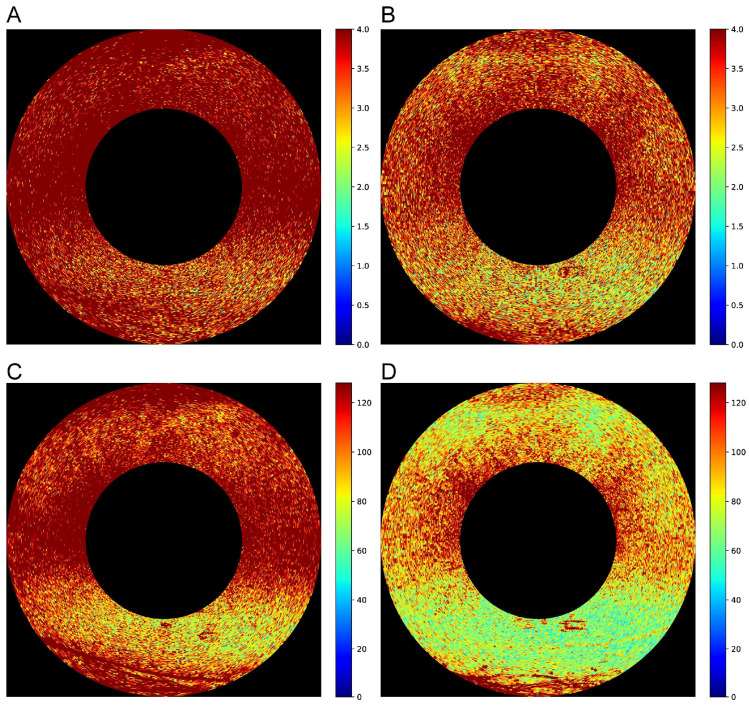
Group-level mean peripheral sharpness heatmaps in the R channel. Pixelwise gradient values within the peripheral annular zone were averaged across all eyes in each group (monofocal: *n* = 32; multifocal: *n* = 33). (**A**) Brenner gradient, monofocal IOL group. (**B**) Brenner gradient, multifocal IOL group. (**C**) Tenengrad gradient, monofocal IOL group. (**D**) Tenengrad gradient, multifocal IOL group. The color scale is derived from the 75th percentile of pooled pixelwise values across all 65 images. Warmer colors indicate higher local sharpness values.

To characterize the spatial distribution of peripheral sharpness at the group level, mean Brenner gradient and Tenengrad gradient heatmaps were generated for the monofocal and multifocal IOL groups (Figure 2). For each eye, pixelwise gradient values were computed within the mid-peripheral annular zone (1.5–3.0 times the fovea–disc distance) and transformed into polar coordinates centered on the fovea, using the fovea–disc distance as an internal scaling reference. This normalization allowed spatial averaging across eyes with varying image sizes and anatomical proportions. Group-level mean heatmaps were then computed by averaging the polar-transformed maps across all eyes within each group. A common color scale was derived from the 75th percentile of pixelwise values pooled across all 65 images for each metric to enable direct visual comparison between groups.

The mean heatmaps revealed consistently lower gradient magnitude across the annular zone in the multifocal group compared with the monofocal group for both metrics, presented as broader cool-colored regions. This group-level spatial pattern corroborates the quantitative findings and suggests that the reduction in peripheral R channel sharpness in multifocal eyes is spatially distributed rather than focal.

### 3.4. Subgroup Analysis Among Multifocal IOL Types

**Figure 3:**
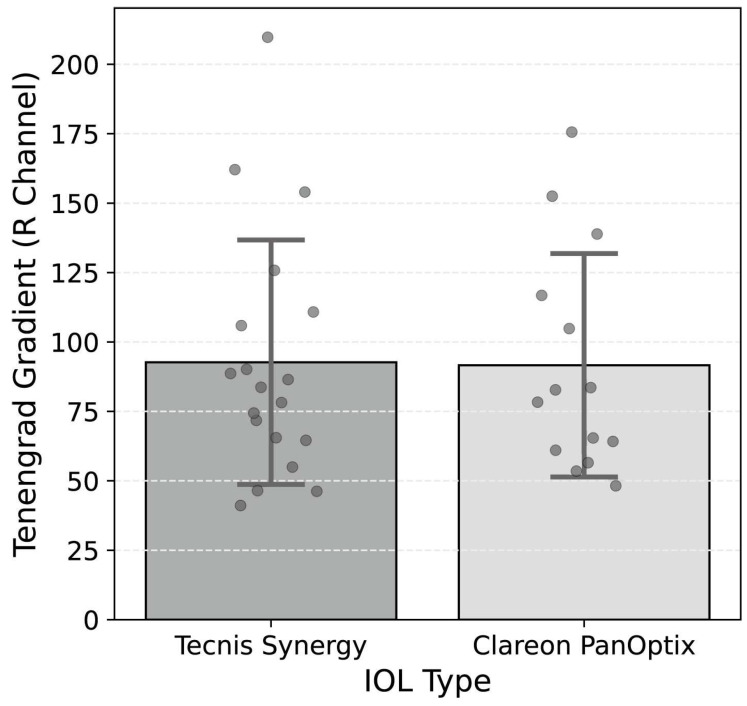
Comparison of peripheral image sharpness across multifocal IOL designs. Mean Tenengrad scores in the red (R) channel with standard deviation are shown for hybrid multifocal and quadrifocal subtypes. No significant difference was observed between groups (*p* > 0.05, Mann–Whitney U test).

A subgroup analysis was performed to compare peripheral sharpness scores between the two predefined multifocal IOL subgroups using Tenengrad in the R channel, which showed significant intergroup differences in the previous analysis. As illustrated in Figure 3, the hybrid multifocal group (92.71 ± 44.03) exhibited a trend toward higher mean Tenengrad scores compared with the quadrifocal group (91.63 ± 40.24); however, this difference did not reach statistical significance (*p* > 0.05, Mann–Whitney U test). The overall distribution patterns were similar between subgroups, with overlapping interquartile ranges and comparable variability.

## 4. Discussion

This study quantitatively compared peripheral ultra-widefield fundus image sharpness between eyes implanted with monofocal and diffractive multifocal IOLs using three established sharpness metrics. Brenner and Tenengrad showed significantly lower peripheral sharpness in the R channel in the multifocal group, whereas no significant differences were observed in the G and composite channels. Laplacian did not demonstrate significant intergroup differences in any channel. These findings indicate that the observed reduction was wavelength-specific and primarily detected by first-derivative–based metrics.

The reduction in peripheral R channel sharpness in multifocal eyes may arise from the optical characteristics of diffractive IOLs [16]. In a conventional trifocal design, the 0th, +1st, and +2nd diffraction orders (m) are utilized to generate distance, intermediate, and near foci, respectively [17,18]. In this configuration, the 0th order provides distance vision through the high-power refractive carrier lens, forming an image of distant objects. Diffractive multifocal IOLs are typically designed based on a green wavelength [17,19]; therefore, the green (G) channel may not demonstrate substantial differences compared with monofocal IOLs, as retinal imaging is formed at optical infinity for distance vision. Positive diffraction orders introduce longitudinal chromatic aberration (LCA) with a sign opposite to that of the cornea and the refractive carrier lens [16,20]. However, at the distance focus (m = 0), LCA remains unaffected [20]. Consequently, the red (R) channel may not show a significant difference compared with monofocals in terms of LCA at the central distance focus [21]. In the peripheral retina, however, transverse chromatic aberration (TCA) becomes more pronounced, which is hypothesized to contribute to increasing differences between multifocal and monofocal IOLs in the R channel toward the periphery [22,23]. This hypothesis may partly explain why significant reduction was detected in the R channel but not in the G and composite channels.

Subgroup analysis among multifocal IOL types did not reveal significant differences in peripheral R-channel sharpness. The Synergy IOL employs the +1st and +2nd diffraction orders—rather than the 0th and +1st orders—to generate distance and near foci, thereby compensating for LCA at the distance focus [24]. Under this configuration, one might expect increased blur in the R channel due to LCA. Nevertheless, no significant difference was observed between Synergy and other multifocal IOLs. This finding may suggest that, in the periphery, the effect of TCA outweighs that of LCA; however, further investigation is required to clarify this mechanism.

Notably, the sharpness reduction was identified within the predefined peripheral annular region. This peripheral predominance is consistent with off-axis optical behavior, as peripheral rays enter the IOL at steeper angles and may be more sensitive to diffractive energy redistribution than central rays [22,25]. The absence of significant differences in Laplacian suggests that second-order high-frequency components were largely preserved. In contrast, Brenner and Tenengrad detected subtle peripheral edge blurring, reflecting the expected sensitivity of gradient-based focus measures [26].

The spatial distribution of sharpness did not exhibit a purely concentric pattern, as might be expected from optical considerations alone. Instead, higher sharpness values were frequently localized along retinal structures, particularly vascular features [27]. This observation suggests that pixelwise sharpness metrics may be influenced not only by optical factors but also by underlying retinal anatomy. The multilayered structure of the retina and variations in local reflectivity may contribute to spatial heterogeneity in measured sharpness, potentially through differential scattering or differential signal intensity across tissue types [28].

An important baseline characteristic was that patients implanted with multifocal IOLs were significantly younger than those receiving monofocal IOLs. This age difference likely reflects real-world lens selection patterns, as multifocal IOLs are typically offered to younger patients seeking spectacle independence, whereas monofocal IOLs are more commonly selected in older individuals. Because aging is generally associated with reduced contrast sensitivity [29,30], this demographic difference would theoretically bias results toward lower sharpness in the monofocal group. However, despite being significantly younger, the multifocal group demonstrated lower peripheral R channel sharpness, supporting the interpretation that the observed difference is more plausibly related to IOL optical design rather than age alone.

Another notable observation was that monofocal eyes tended to show higher upper-end sharpness outliers. Monofocal IOLs concentrate incoming light into a single focal point, which may maximize peak gradient responses under optimal optical conditions. In contrast, diffractive multifocal IOLs divide light energy among multiple focal points [18], potentially reducing the likelihood of extreme peak sharpness values even when overall image quality remains comparable. Thus, the broader upper distribution in the monofocal group likely reflects differences in optical energy allocation rather than measurement variability.

From a clinical standpoint, the magnitude of sharpness reduction was modest and localized. Although the reduction in R-channel sharpness reached statistical significance, this finding was confined to a single channel and metric pair; it was not observed in the composite channel used for routine clinical review. This suggests the effect represents a measurable optical phenomenon rather than a clinically validated reduction in diagnostic accuracy. Studies directly assessing detection of peripheral lesions (e.g., masked grading of retinal tears or vascular lesions) would be needed to establish whether this effect meaningfully affects diagnostic confidence.

This study has several limitations. The sample size was relatively small, which may limit generalizability. Additionally, the monofocal comparator group consisted exclusively of enhanced monofocal IOLs (Tecnis Eyhance), as no eligible patients implanted with simple monofocal IOLs were identified during the study period at our institution. Whether the observed findings generalize to comparisons with simple monofocal IOLs remains to be investigated. Ocular biometric parameters such as axial length, refraction, and postoperative visual acuity were not systematically recorded or controlled for, and may represent additional sources of variability in image sharpness that could not be accounted for in the current analysis. Pupil size at the time of image acquisition was not recorded and could not be adjusted for; smaller pupils can reduce higher-order aberrations and could theoretically modulate the observed differences. Furthermore, all images were acquired under non-mydriatic conditions with central fixation, reflecting routine outpatient UWF screening. Dilated fundus photography or eccentric-fixation imaging could provide a more direct assessment of the peripheral retina and may be more sensitive to genuine pathology; comparison of mydriatic and non-mydriatic conditions would be a valuable direction for future work. Eyelash artifacts within the peripheral analysis zone could not be reliably excluded, as their appearance on UWF imaging is visually similar to retinal structures and could not be robustly distinguished even by manual review, representing a residual source of measurement noise that may have influenced sharpness values in a subset of images. Moreover, the annular region of interest contains relatively sparse high-contrast structures—predominantly small-caliber retinal vessels—which constitute the principal source of sharp edges available to the sharpness algorithms. Because vessel density and distribution are not perfectly uniform across images, sharpness metrics may be differentially sensitive to true optical blur versus local vascular content, which may partly explain why the Laplacian metric did not detect the group difference identified by the first-derivative Brenner and Tenengrad metrics. Although these analyses were specified a priori, the possibility of type I error due to multiple comparisons across three metrics and three channels cannot be entirely excluded. Finally, because we analyzed quantitative image sharpness metrics rather than direct diagnostic performance outcomes, the clinical implications should be interpreted with caution.

## 5. Conclusions

Diffractive multifocal IOL implantation was associated with a statistically significant but spatially localized reduction in peripheral R channel sharpness. This localized effect likely reflects intrinsic optical energy redistribution inherent to diffractive lens designs rather than generalized image degradation. Overall, composite channel quality remained comparable between groups, suggesting limited clinical impact under routine imaging conditions. Further prospective studies incorporating larger cohorts and diagnostic performance outcomes would help clarify the broader clinical relevance of these observations.

## Figures and Tables

**Table 1:** Participant Demographics and Intraocular Lens Distribution.

		Monofocal (*n* = 32)	Multifocal (*n* = 33)	*p* Value
Age (yrs) *		70.84 ± 6.03	60.76 ± 12.83	<0.01 ^†^
Sex (Male:Female)		14:18	12:21	0.62 ^‡^
Laterality (OD:OS)		16:16	19:14	0.62 ^‡^
IOL Model	Tecnis Eyhance	*n* = 32	—	—
	Tecnis Synergy	—	*n* = 19	—
	Clareon PanOptix	—	*n* = 14	—

* Mean ± SD. ^†^ Mann–Whitney U test. ^‡^ Fisher’s exact test.

## Data Availability

The data supporting the findings of this study are available from the corresponding author upon reasonable request.
